# Ancient and Archaic Genomes

**DOI:** 10.3390/genes12091411

**Published:** 2021-09-13

**Authors:** Stefania Vai, Martina Lari, David Caramelli

**Affiliations:** Department of Biology, University of Florence, 50122 Florence, Italy; martina.lari@unifi.it (M.L.); david.caramelli@unifi.it (D.C.)

The first data obtained from ancient DNA samples were published more than thirty years ago. During this time, methodological innovations signed by polymerase chain reaction (PCR) first, and next-generation sequencing (NGS) later allowed researchers to understand the molecular features of ancient DNA and to reconstruct even whole genomes from organisms that lived in the past. Dedicated facilities, specific experimental procedures, and bioinformatics pipelines are required to obtain reliable ancient DNA data taking into account preservation, degradation, and contamination issues [1]. Thanks to the already consolidated knowledge of DNA degradation processes and to the high-throughput sequencing methodologies, thousands of archaeological specimens (mostly bones and teeth, but also hairs, mummified soft tissues, coprolites, and vegetable materials) have been analyzed thus far at a genomic level, providing information for a deep understanding of evolutionary processes, anthropological questions, and archaeological reconstructions. Indeed, ancient DNA studies, providing direct data from the past, offer us the possibility to observe genetic variation through time, allowing us to go beyond the limits of inferential approaches relying on modern data only.

This Special Issue titled “Ancient and Archaic Genomes” collects original research articles that present different methods and aspects of the paleogenetic research applied to anthropological, archaeological, and historic questions. Interestingly, specific regional contexts and cultural aspects previously poorly studied from a genetic point of view are here investigated.

Through an experimental strategy based on PCR and Sanger sequencing, Gînguță and colleagues [2] investigated the maternal genetic diversity of medieval individuals from Transylvania (Romania). The mitochondrial DNA control region of 13 individuals from the Feldioara necropolis (12–13th century) was analyzed and compared with historical and modern populations. A high genetic variability was found, with all the individuals characterized by a different mitochondrial lineage, mostly carrying West Eurasian haplogroups and with a possible contribution related to the arrival of Hungarian conquerors at the end of the 9th century.

Thanks to NGS methodology, even complete mitochondrial genomes can be reconstructed, increasing the informative power of this uniparental marker. In this Issue, two contributions based on the analysis of complete or almost complete mitogenomes are dedicated to ancient human samples, while one article is focused on animal specimens.

Kusliy et al. [3], indeed, present a study on the Mongolian horse, one of the most ancient horse breeds. They obtained nearly complete mitochondrial genomes from six samples of the Khereksur and Deer Stone cultures (late 2nd to first third of the 1st millennium BC) and from the Xiongnu culture (1st century BC to 1st century AD). A phylogenetic analysis revealed genetic continuity between the Mongolian horse populations of the three ancient cultures, and a comparison with other ancient, historical, and modern mitogenomes of horses indicated close relationships with indigenous breeds of the Middle East, Eastern and Central Asia, and the Mediterranean region.

In the context of the study of past human population dynamics, Fontani and colleagues [4] present the first ancient molecular data from the Calabria region (southwestern Italy): an important contribution to fill the gap in the limited knowledge of prehistoric genetic variability in the Italian Peninsula. Two mitochondrial genomes were obtained from a Middle Bronze Age mass grave in a karstic cave called Grotta della Monaca. Possible affinities with other Italian and European ancient populations are highlighted through a phylogenetic analysis, providing a starting point for the study of population dynamics and migrations in Southern Italy.

Another research article presenting data on whole mitochondria is focused on 10–11th century remains in the Carpathian Basin. Maár et al. [5] analyzed more than 200 new mitogenomes for the commoner population in order to compare them with the data available for the immigrant elite including conquering Hungarians. Phylogenetic analysis and haplogroup- and sequence-based methods provide a first description of this population and the relationships with other ancient Eurasian groups, highlighting differences and possible admixture with the eastern immigrants.

A more accurate picture of the population dynamics in these European regions, firstly described through mitochondrial genetic variability, might be provided by future genome-wide studies. In fact, mitochondrial data alone provide a partial view of the genetic history of a population. A greater contribution than NGS technology provided to ancient DNA studies is represented by the possibility to obtain reliable autosomal data that can reveal more details not only at a population but also at an individual level.

Autosomal data are often obtained in ancient degraded samples through a target enrichment approach focused on genotyping a set of informative SNPs. Based on this approach, mitochondrial and nuclear SNP data are presented in a case study showing how paleogenomic analysis of ancient human remains can provide information useful for individual characterization and archaeological reconstruction [6]. The minimum number of individuals, their sex and phenotypic traits, and the kin relationships between them were estimated for samples found in a complex scenario from the Neolithic time in Poland: a multiple secondary burial where skeletal remains of several individuals were intentionally fragmented and mixed. The analysis of molecular damage also highlighted the presence of modern remains added in historical time to the burial boundary, helping to clarify the reconstruction of the exploitation of this site through time.

The SNP selection strategy is often the only way to obtain nuclear genome data for highly degraded samples with a low content of endogenous DNA. However, when allowed by DNA preservation, a whole-genome sequencing (WGS) strategy allows performing sophisticated analysis that helps to understand past demographic scenarios and infer details about populations’ structure and interactions.

Vizzari et al. [7] tested the two main out-of-Africa hypotheses through an approximate Bayesian computation approach based on the random forest algorithm. It is still under debate, indeed, whether anatomically modern humans (AMH) left Africa through a single dispersion event or in two main waves (first toward southern Asia and Australo-Melanesia, and later through a northern route). By comparing simulated data with real genomic variation observed by analyzing high-coverage genomes of archaic (Denisovan and Neandertal) and modern populations, they show that a model of multiple dispersals is four-fold as likely as the alternative single-dispersal model. Modern Australo-Melanesians derive from a migration from Africa that may have occurred around 74,000 years ago, while Eurasians derive from a second dispersal event dating back to around 46,000 years ago.

We believe that this Special Issue, presenting different methodological approaches and applications, will be a useful resource for both students and young researchers who are interested in ancient DNA studies.
